# Clinical Application of AMH Measurement in Assisted Reproduction

**DOI:** 10.3389/fendo.2020.606744

**Published:** 2020-12-09

**Authors:** Hang Wun Raymond Li, Scott M. Nelson

**Affiliations:** ^1^ Department of Obstetrics and Gynaecology, The University of Hong Kong, Pokfulam, Hong Kong; ^2^ School of Medicine, University of Glasgow, Glasgow, Scotland, United Kingdom; ^3^ NIHR Bristol Biomedical Research Centre, Bristol, United Kingdom; ^4^ The Fertility Partnership, Oxford, United Kingdom

**Keywords:** anti-Müllerian hormone, assisted reproduction, *in vitro* fertilization, intrauterine insemination, ovarian response, gonadotropin dosing

## Abstract

Anti-Müllerian hormone reflects the continuum of the functional ovarian reserve, and as such can predict ovarian response to gonadotropin stimulation and be used to individualize treatment pathways to improve efficacy and safety. However, consistent with other biomarkers and age-based prediction models it has limited ability to predict live birth and should not be used to refuse treatment, but rather to inform counselling and shared decision making. The use of absolute clinical thresholds to stratify patient phenotypes, assess discordance and individualize treatment protocols in non-validated algorithms combined with the lack of standardization of assays may result in inappropriate classification and sub-optimal clinical decision making. We propose that holistic baseline phenotyping, incorporating antral follicle count and other patient characteristics is critical. Treatment decisions driven by validated algorithms that use ovarian reserve biomarkers as continuous measures, reducing the risk of misclassification, are likely to improve overall outcomes for our patients.

## Introduction

One major clinical area where anti-Müllerian hormone (AMH) measurement has carved out a clear niche is in assisted reproduction. Over the recent two decades, there have been a growing number of observational studies and randomized controlled trials (RCTs) exploring AMH’s role in assessing ovarian reserve, predicting ovarian response and outcomes to treatment, and clarifying its utility for individualizing treatment strategies in women undergoing assisted reproduction treatment (ART) including intrauterine insemination (IUI) with controlled ovarian stimulation or *in vitro* fertilization (IVF). These clinical applications are based on AMH exhibiting strong correlations with the primordial follicle count (1), the ultimate parameter that represents the conceptual ovarian reserve, as well as the later stages of follicular development that are responsive to gonadotropins and constitute the functional ovarian reserve (2–4). The widespread clinical adoption of AMH has been further enhanced through the ease of scalability of serum/plasma testing, the availability of high precision automated assays, that it can be measured at any part of the menstrual cycle, that small fluctuations observed within and across cycles have been shown not to be clinically important (5–11), and that measurements determined in the months leading up to the index stimulation cycle can accurately guide assisted reproduction treatment decisions. In this review, we summarize the recent evidence underpinning the use of AMH in ART, including how AMH can inform the overall prognostic phenotype and individualize treatment decisions, while highlighting the areas that continue to require further exploration.

## Initial Observations

### AMH as a Continuous Measure

The AMH concentration that we receive from that initial blood test is like many biological measures lying on a continuum, from very low at one extreme to very high at the other. However, historically studies have primarily focused on identifying individual threshold values to categorize different types of prognosis and stratify treatments (12, 13). In clinical practice it is often helpful to label individuals as having or not having an attribute, such as a potential poor responder, depending on the value of a continuous variable like AMH. However, the dichotomization of continuous variables leads to several problems (14). Importantly much information is lost, so the statistical power and ability to demonstrate an association with the outcome is substantially reduced. Secondly the extent of the variation in outcome between groups may be underestimated, such that individuals close to but on opposite sides of the threshold are characterized as being very different rather than being very similar. Thirdly, using two groups conceals any non-linearity in the relation between the variable and outcome. Lastly, the use of apparently “optimal” cutpoint (usually that giving the minimum P value) runs a high risk of a spuriously significant result; the difference in the outcome variable between the groups will be overestimated, perhaps considerably, and the confidence interval will be too narrow. In ART these issues are not unique to AMH and apply to many routine indices like antral follicle count (AFC), sperm counts, and endometrial thickness which are all continuous measures yet we continue to dichotomize them rather than treat them as continuous variables to enable greater variability in outcomes to be explained. Confirmation of the value of treating AMH as a continuous measure, enabling greater explanation of variability, has recently been shown in a RCT (15). Simple categorization has a role, but as we become more sophisticated in our understanding of the strengths and weaknesses of AMH we propose that we should consider AMH as biology intended—a continuous measure of the functional ovarian reserve.

### Ovarian Reserve Markers Should Not Be on the Causal Pathway When Estimating Strength of Associations With Outcomes

Assessment of the true strength of the correlation between an exposure (such as AMH) and an outcome (for example oocyte yield) requires that the exposure in no way influences the treatment pathway which results in the outcome. Unfortunately for many of the studies that blindly evaluated the correlation of AMH with outcomes (16, 17), also measured and acted upon the antral follicle count (AFC), which is itself strongly correlated with AMH due to the granulosa cells of the smaller antral follicles being the primary source of AMH (4). By altering the stimulation strategy or dose based on the AFC or other linked factors like age, the researchers will have introduced systematic bias which can lead to an overestimation of the strength of the correlation, which will apply to the primary marker used such as AFC and to a lesser degree the inter-related markers like AMH. The only way the strengths of the association can be truly evaluated is if all patients are treated identically and the researchers are blind to the initial ovarian reserve biomarkers (18). Such a study design is often only seen in randomized controlled trials (19, 20), rather than observational studies which are frequently used to assess and report the relative merits of different biomarkers. It is in this context of multicenter large scale RCTs with strictly defined protocols, where a true indication of the performance and limitations of biomarkers such as AFC and AMH can be observed.

### Role of AMH Measurement In IUI With Controlled Ovarian Stimulation

Controlled ovarian stimulation and IUI can be a first-line treatment for unexplained infertility as well as infertility due to mild male factor or endometriosis. In this context IUI may commonly be coupled with controlled ovarian stimulation by gonadotropin or oral anti-estrogen drugs which may correct subtle problems of ovulation, slightly increase the number of oocytes available for fertilization, and enhance the accuracy of timing of insemination (21, 22).

For AMH to be of value in this context, we anticipate it would identify which patients would benefit from stimulated IUI rather than proceeding directly to IVF, identify an appropriate initial stimulation strategy for example exogenous gonadotropins or aromatase inhibitors, and/or identify the likely prognosis to manage patient expectations regarding the likelihood of success. Unfortunately, although AMH contributing to all three aspects may be aspirational there have been a limited number of studies evaluating the role of AMH in IUI management. Historically, studies focused on the association of pretreatment AFC with pregnancy outcomes, with differing conclusions (23–25). The first study on the role of serum AMH in predicting treatment outcome after ovarian stimulation using gonadotropins was reported in 2010 in 243 women undergoing IUI (26). In this study, baseline AFC was used to alter the dose of starting gonadotropin (ovarian stimulation was achieved with hMG or recombinant FSH starting at 150 IU/day, except for those with AFC ≥10 or polycystic ovaries, where it was started at 100 and 75 IU/day, respectively). In women who attained live birth either in the first treatment cycle or cumulatively over three treatment cycles, their pretreatment AMH was significantly higher than those who did not (median AMH 3.47 ng/ml vs. 2.04 ng/ml). Furthermore, AMH remained a significant predictor on the likelihood of cumulative live birth after controlling for age and body mass index of the women in a logistic regression model. Others have subsequently reported similar associations between higher AMH and higher success rates (27–29). To date validation of optimal treatment strategies (30) or of retrospectively derived algorithms (31) have however been limited and would be the next step for confirmation of a more widespread role of AMH in stimulated IUI programs.

### Role of AMH Measurement In IVF

In IVF program, numerous studies have explored the role of serum AMH measurement in predicting ovarian response to gonadotropin stimulation, individualizing treatment pathways to improve efficacy and safety, and lastly predicting overall treatment success (12, 13, 18, 32). Given the biological premise of AMH as a functional ovarian reserve marker, it is not surprising that it is these first two areas where AMH has made the greatest contribution.

### Prediction of Suboptimal or Excessive Ovarian Response

Ovarian stimulation forms an integral part in modern IVF programs. Multiple follicle development and aspiration, and hence collection of multiple oocytes, helps to increase the efficiency of the treatment program. It has been reported that a higher oocyte yield up to around 15 was associated with higher live birth rate in the fresh treatment cycle (33) as well as higher cumulative live birth rate following the fresh and all frozen-thawed embryo transfers after one IVF cycle (34).

It is now widely established that AMH and AFC are the currently best available predictors of ovarian response and its associated extremes; poor and excessive ovarian response (35–37). Accepting the limitations noted above on using observational studies, an individual patient data (IPD) meta-analysis assessing prediction of excessive ovarian response included 57 studies with 4,786 women and concluded that both AMH and AFC exhibited similar and reasonably good performance in predicting excessive ovarian response in isolation. The area under the receiver operating characteristic (ROC) curve for AMH and AFC was 0.81 and 0.79 respectively (16). Although the combination improved the area under the curve marginally to 0.85, inclusion of additional covariates such as age or FSH did not improve the prediction further. A second IPD meta-analysis reported on the prediction of poor ovarian response (17). It included data from 28 studies with 5,705 women undergoing IVF treatment. Again, both AMH and AFC had similar and reasonably good performance in predicting poor ovarian response on their own, with an area under the ROC curve of 0.78 and 0.76 respectively, and once again combining the two or adding age did not significantly improve the prediction. However, as noted for almost all of the studies included in these meta-analyses, AFC was known prior to commencing ovarian stimulation and used to modify the dose which might have led to an overestimation of the strength of the association of AFC.

In the context of predicting suboptimal ovarian response, AMH and AFC were among the criteria used to define or predict poor ovarian responders in the Bologna criteria (38) and more recently the Poseidon classification (39). Although the Poseidon criteria has been proposed as an attempt at defining a more homogeneous population, heterogeneity remains. For example, the thresholds for AMH and AFC are not aligned with respect to established correlations between these two indices (4). For treated patients there is no agreed consensus on the nature of the previous stimulation strategy. Lastly patients are dichotomized to either <35 or ≥35 years of age, despite the non-linear relationship with oocyte aneuploidy.

An extrapolation of the ability to predict ovarian response is the individualization of the ovarian stimulation regimen in the treatment naïve patient, particularly if aiming for a fresh embryo transfer. In this context achieving an optimal oocyte yield while minimizing the risk of ovarian hyperstimulation syndrome (OHSS) is paramount. Our initial suggestion of AMH-driven algorithms to determine the initial gonadotropin dose and regimen of ovarian stimulation (40, 41) have now been confirmed in several RCTs, with the largest (n=1326) assessing the efficacy and safety of follitropin delta (15). In this RCT, the follitropin delta dose was based on the individual women’s serum AMH level and body weight and compared with follitropin alpha at 150 IU with subsequent step up or down according to ovarian response. The two treatment arms had similar mean oocyte yield and live birth rates, and yet the follitropin delta arm had significantly lower rates of suboptimal or excessive response. A recent Cochrane meta-analysis concluded that although individualized dosing of gonadotropin based on ovarian reserve markers might not influence the rate of ongoing pregnancy or live birth compared to standardized dosing, it could reduce the incidence of moderate-to-severe OHSS by prompting the use of a reduced dose of gonadotropin in predicted high responders (42).

As current AMH assays give different numerical results without any universal standardization (43, 44), and that reported studies were performed using different assays, it is not possible to combine the available data to determine cut-offs for predicting excessive or suboptimal ovarian response. Similarly, although historical definitions including the Bologna criteria defined poor ovarian reserve as an AFC of below 5 to 7, or AMH level of below 0.5–1.1 ng/ml, while the Poseidon classification adopted an AFC of 5 or AMH of 1.2 ng/ml as the cut-offs for defining it, it should be noted that these were based on previous studies using different assay methods and hence there may be problems to adopt these apparently simple thresholds universally.

### Prediction of Pregnancy or Live Birth in IVF Treatment

Despite the good performance of AMH and AFC in predicting ovarian response, most studies, however, consistently showed that just like age, AMH and AFC were poor overall predictors of pregnancy or live birth in the fresh IVF cycle (17, 32, 45). The summary ROC curves derived from the individual patient data meta-analysis by Broer et al. (17) for prediction of ongoing pregnancy confirmed the limited role of AMH, AFC and age, or their combinations, with an area under the ROC curve of less than 0.6. Focusing on AMH and live-birth, a meta-analysis on 13 studies found an area under the ROC curve of 0.61 (95% confidence interval 0.56–0.65) of confirming the limited contribution that AMH in isolation would have for prognostication of overall live-birth (32).

All these studies were limited by focusing on the rate of pregnancy or live birth in the fresh IVF cycle only. In modern-day IVF programs, embryo cryopreservation constitutes an increasingly important part, and hence the cumulative live birth rate from the fresh and all frozen-thawed embryo transfer (FET) cycles combined would be more informative and meaningful than the outcome of the fresh cycle alone (46). A retrospective analysis evaluating the role of baseline AMH in predicting cumulative live birth from the fresh IVF cycle plus all subsequent FET cycles derived from that stimulated cycle was first reported in 2013 (3). It included 1,156 women undergoing the first IVF cycle in a single center treated under the long GnRH agonist protocol or GnRH antagonist protocol. It suggested that the cumulative live birth rate followed a gradual rising trend with serial increase in serum AMH or AFC over a continuum instead of showing an abrupt change at any threshold value. However, both parameters had only modest performance, which was not better than the women’s age alone, in predicting the absolute occurrence of cumulative live birth as demonstrated by the ROC curves (area under the curve being 0.646, 95% CI 0.616–0.675). After controlling for the women’s age and the number of embryos replaced, both serum AMH and AFC were not significant independent predictors of live birth in the fresh IVF cycle nor cumulative live birth suggesting that their association with overall livebirth was through the number of oocytes and thereby number of embryos available to transfer. Another recent study in 9,494 Chinese women similarly demonstrated that increasing AMH up to 5–7 ng/ml predicted better cumulative live birth rate in IVF and that it was mainly through the association with oocyte yield (47).

As for women with predicted poor ovarian reserve, a recent retrospective analysis on 825 IVF cycles showed that the live birth rate decreased through Poseidon groups 1, 3, 2, and 4 in order (48). It implies that both AMH or AFC as well as age have an impact on the prediction of live birth. It is worth to note that in the study by Li et al. (3), women with serum AMH <0.5 ng/ml still had a cumulative live birth rate of 27%, and cumulative live birth did occur in women with AMH as low as 0.15 ng/ml. Another secondary analysis on women in the OPTIMIST study, a prospective observational study on 551 women with predicted low prognosis, showed that those in Poseidon 4 group (older women with low ovarian reserve) still had conservative and optimistic cumulative live birth rates of 37% and 41% respectively over 18 months of treatment (49).

Collectively all of these data suggest that a patient at any age with a higher AMH has an overall better prognosis. However, due to the limitations of its predictive performance, a threshold value should not be used to deny women from attempting ART, nor to be too pessimistic regarding prognosis based solely on an AMH value.

### Comparing AMH Versus AFC in Predicting Ovarian Response

Although differences in performance characteristics of AMH and AFC have been reported in several multi-center RCTs (18), direct head-to-head performance comparison of AMH- or AFC-based dosing algorithms has been more limited. Specifically, two RCTs have compared the performance of a serum AMH or AFC algorithm in predicting ovarian response in an IVF program, with both concluding that there were no significant differences in the proportion of cycles attaining desired ovarian response when the gonadotropin dosing algorithm was determined based on either AMH or AFC (50, 51). In the first study, 348 Vietnamese women were treated with a long GnRH agonist protocol, and 35.2% versus 28.4% of cycles attained the desired response when the AMH-based and AFC-based algorithms were adopted respectively (p>0.05), although the incidence of hyper-response was significantly lower in the AMH group (8.6%) compared to the AFC group (17.4%) (50). In the second study, 200 participants from Hong Kong were treated on a GnRH antagonist protocol (51). There were no significant differences in the proportion of cycles with desired response between the AMH-based and AFC-based groups (49.0% versus 54.0%, p>0.05), or the number of oocytes retrieved or the follicular output rate. However, significantly more women required an increase in their gonadotropin dose in the AMH group compared to the AFC group. These findings suggest that clinicians who choose to use these specific published algorithms and treatment strategies would obtain equivalent results whether they use AMH or AFC. However, this conclusion of equivalence does not extend to other untested algorithms or equate to overall equivalence for treatment decision making.

### Discordance Between AMH and AFC in Prediction of Ovarian Response

For most women AMH and AFC will be similar, but discordances can occur with extreme disagreements the most concerning and difficult to interpret clinically. A retrospective analysis on 1,046 women assessed the discordance between AMH and AFC, by using the 25^th^ and 75^th^ centiles of AMH (1.4 and 5.3 ng/ml) and AFC (6 and 14) respectively as the thresholds (52). In these analyses only 4 patients exhibited a high AMH but a low AFC and conversely 1 patient exhibited a high AFC and low AMH. Simple categorization may however over emphasize apparent milder discordances, for example in the above study an AMH of 1.3 ng/ml and an AFC of 7 would be recorded as discordant, but clinically many would perceive as equivalent with a similar response anticipated. In the trial by Li et al. (51), among the 200 enrolled women, 26.5% showed discordance between categorization based on AMH or AFC in the pre-treatment cycle (κ=0.560), with an overall discordance rate of around 30%. In women who were discordant in AMH and AFC categories, those having higher AMH within the same AFC quartile had significantly higher oocyte yield and cumulative live birth rate, and the ovarian responsiveness was intermediate between those where AMH and AFC were concordant on either the high or low end (52). Applying to clinical scenarios where AMH and AFC categories are discordant, it is reasonable to suggest an intermediate dose of gonadotropin between that assigned for the high and low ends. Nonetheless, such a recommendation will require verification in prospective trials.

In view of such discordant scenarios, it has been proposed that a more holistic phenotype which incorporates AMH, AFC and age can be combined into a composite score for the purpose of ovarian response prediction. The ovarian response prediction index (ORPI), calculated as the product of AMH level (ng/ml) and AFC divided by age of the woman (years), was first reported by Oliveira et al. (53). The original study showed that ORPI had good prediction on oocyte yield, and the same group subsequently also showed that using ORPI for individualization of the ovarian stimulation regimen resulted in elimination of OHSS in their center (54). A retrospective analysis on 285 women stimulated with a standardized initiation dose using corifollitropin alpha in the GnRH antagonist protocol confirmed that ORPI was significantly correlated with the oocyte yield (55). ROC curve analysis revealed that the area under the curve for ORPI was comparable to AMH alone and significantly higher than AFC alone for prediction of excessive response, while it was significantly higher than that of AMH or AFC alone for prediction of suboptimal response. In contrast in the phase II derivation of the follitropin delta algorithm, the inclusion of either or all of age, FSH, or AFC did not increase explanation of the variance by ≥5% above what was initially observed for just bodyweight and AMH. Therefore, although a composite index of ovarian reserve biomarkers may be worth further exploration to try to reduce the unexplained variance in ovarian response in future trials, its overall contribution may be limited and would require systematic and timed scanning.

### Timing of AMH Assessment Prior to IVF

A number of studies have reported inter-cycle fluctuations of AMH level, and yet the absolute magnitudes of these fluctuations are small and may have limited clinical importance. It was shown that when AMH was measured one month prior to IVF as well as at the start of ovarian stimulation, there was moderate concordance between AMH categorization measured in the pre-treatment versus the stimulation cycle (κ = 0.573) (51). Similarly, an analysis of 1326 women in the three months leading up to an index cycle suggested strong correlations (r=0.92), with no systematic variation across the menstrual cycles (56). Others have also shown using different gonadotropins that it can be used in advance of the index cycle for prediction of response (13, 57). Hence, although the assessment of AMH can be performed on any day of the cycle in the months preceding ovarian stimulation, as for any response prediction the accuracy will be greatest if it is measured in the index cycle immediately prior to commencing gonadotropins.

### Prediction of Embryo Quality

There are contrasting data reported on the role of AMH in predicting embryo quality. While some studies revealed that serum AMH was not significantly associated with morphokinetic embryo quality as assessed by time-lapse imaging (58, 59), there were reports that the oocyte-specific AMH concentration in follicular fluid had good prediction on embryonic development and live birth (60, 61). It is interesting to further explore the functional relationship of follicular and serum AMH with oocyte competence, embryo euploidy and its role in embryo selection.

## Concluding Remarks

AMH has evolved as a useful tool for the assessment of the functional ovarian reserve and prediction of ovarian response, with performance at least equivalent to or better than AFC. However, just like AFC or age, its ability to predict live birth both in the fresh cycle and cumulatively taking into account all embryos derived from the same index stimulation cycle is limited, and primarily stem from its relationship with the oocyte and hence embryo yield. We propose that we no longer need to debate on which biomarker is best, but rather accept that we can utilize all of the information at our disposal to characterize the baseline phenotype and likely response and modify our treatment strategies accordingly. The use of both AMH and AFC, as continuous measures, combined with other patient characteristics in validated algorithms will reduce the risk of misclassification and is likely to improve overall outcomes for our patients.

## Author Contributions

HL and SN conceived, wrote, and approved the manuscript. All authors contributed to the article and approved the submitted version.

## Conflict of Interest

SN reports personal fees from Access Fertility, personal fees and non-financial support from Ferring, personal fees and non-financial support from Merck, personal fees from Coopers Genomics, personal fees and non-financial support from Ferring Pharmaceuticals, personal fees from The Fertility Partnership, grants, personal fees and non-financial support from Roche Diagnostics, personal fees from Modern Fertility, other from Delivery I, outside the submitted work.

The remaining author declares that the research was conducted in the absence of any commercial or financial relationships that could be construed as a potential conflict of interest.
